# Enrichment of Deleterious Mutated Genes Involved in Ciliary Function and Histone Modification in Brain Cancer Patient-Derived Xenograft Models

**DOI:** 10.3390/biomedicines11112934

**Published:** 2023-10-30

**Authors:** Hyeongsun Jeong, Hyo Eun Moon, Seongmin Yun, Seung Woo Cho, Hye Ran Park, Sung-Hye Park, Kyungjae Myung, Taejoon Kwon, Sun Ha Paek

**Affiliations:** 1 Department of Biomedical Engineering, Ulsan National Institute of Science and Technology (UNIST), Ulsan 44919, Republic of Korea; 2 Center for Genomic Integrity (CGI), Institute for Basic Science (IBS), Ulsan 44919, Republic of Korea; 3 Department of Neurosurgery, Cancer Research Institute, Seoul National University College of Medicine, Seoul 03080, Republic of Korea; 4 Department of Neurosurgery, Hypoxia/Ischemia Disease Institute, Seoul National University College of Medicine, Seoul 03080, Republic of Korea; 5 Department of Neurosurgery, Soonchunhyang University Seoul Hospital, Seoul 04401, Republic of Korea; 6 Department of Pathology, Neuroscience Research Institute, Seoul National University College of Medicine, Seoul 03080, Republic of Korea; 7 Advanced Institute of Convergence Technology, Seoul National University, Suwon 16229, Republic of Korea

**Keywords:** patient-derived xenograft (PDX), somatic mutations, histone modification, ciliogenesis, brain tumor

## Abstract

Patient-derived xenograft (PDX) models, which can retain the characteristics of original tumors in an in vivo-mimicking environment, have been developed to identify better treatment options. However, although original tumors and xenograft tissues mostly share oncogenic mutations and global gene expression patterns, their detailed mutation profiles occasionally do not overlap, indicating that selection occurs in the xenograft environment. To understand this mutational alteration in xenografts, we established 13 PDX models derived from 11 brain tumor patients and confirmed their histopathological similarity. Surprisingly, only a limited number of somatic mutations were shared between the original tumor and xenograft tissue. By analyzing deleteriously mutated genes in tumors and xenografts, we found that previously reported brain tumor-related genes were enriched in PDX samples, demonstrating that xenografts are a valuable platform for studying brain tumors. Furthermore, mutated genes involved in cilium movement, microtubule depolymerization, and histone methylation were enriched in PDX samples compared with the original tumors. Even with the limitations of the heterogeneity of clinical lesions with a heterotropic model, our study demonstrates that PDX models can provide more information in genetic analysis using samples with high heterogeneity, such as brain tumors.

## 1. Introduction

A brain tumor is one of the hardest tumors to cure, with a 5-year overall survival rate of less than 35%, even though many personalized therapeutic options have been developed [1]. In 2016, the World Health Organization (WHO) started using molecular biomarkers together with traditional histology to classify central nervous system (CNS) tumor types [2]. Since then, the role of molecular biomarkers in CNS tumor classification has gained importance for providing diagnostic information. For example, the fifth edition of the WHO Classification of Tumors of the Central Nervous System (CNS5) incorporates molecular parameters with clinicopathologic phenotypes for the most accurate CNS tumor classification [3].

Large-scale genomic analyses, including The Cancer Genome Atlas project, provide information about molecular markers to group glioblastoma multiforme (GBM) into at least four molecular subtypes depending on genetic, epigenetic, and transcriptional alterations [4,5,6]. More recently, cIMPACT-NOW (the Consortium to Inform Molecular and Practical Approaches to CNS Tumor Taxonomy) has updated practical recommendations on recent advances in CNS tumor classification [7,8,9,10,11]. However, while the importance of molecular biomarkers for CNS tumors is emerging, the significant intra- and inter-tumor heterogeneity of some progressive brain tumor types such as GBM hinders the characterization of mutations critical for tumor progression [12,13].

Patient-derived xenograft (PDX) models are considered a promising platform for personalized cancer research because they can recapitulate the genomic and epigenetic characteristics and microenvironment interactions during the progression of a brain tumor. The Mayo Clinic Brain Tumor PDX National Resource team analyzed the genetic alterations in 83 PDX samples from 24 patient tumors using whole-exome sequencing [14]. They reported that PDX samples preserved most of the genetic driver alterations in the original tumor samples and could even capture common and rare mutations of TERT, EGFR, PTEN, TP53, BRAF, and IDH1 comparable with glioblastoma tissue samples. However, although tumor driver mutations and their expression profiles were concordant between tumors and xenografts, the full list of genetic variations identified in each condition was not reported.

A previous study reported discordance of the detailed mutation profile between xenograft and patient tumors with some sample dependency, ranging from 12% to 64% of shared mutations based on whole-genome sequencing [15]. Another study claimed that some tumor-PDX pairs did not share many somatic mutation profiles as expected, probably due to their hypermutation capability [14]. Although we speculated that the heterogeneity of somatic tumor mutations might cause genetic discordance, the host-specific evolution of xenograft tissues was another possibility [16].

A recent study analyzing 15 trios of tumor-BTIC (brain tumor-initiating cell)-xenograft samples showed that some mutations were shared among the trio samples, but others were only identified in the xenograft model [15]. They further characterized mutations only identified in the xenograft model and found that they were located in genes involved in ciliary or flagellar motility, sensory organ development, and the regulation of synaptic transmission. These studies suggest that cancer genetic studies using PDX models should consider specific selection in the xenograft environment, which has not been thoroughly investigated.

To increase the understanding of these xenograft-specific mutation profiles, we analyzed genome-wide alterations in several brain tumor–PDX models together with their original tumors. Although their histological characteristics were concordant, surprisingly, only a few deleterious somatic mutations overlapped between the PDX samples and the original tumors. By analyzing the PDX-specific enriched mutations, like in the previous report [9,15], we identified mutations in genes related to cilium movement, microtubule depolymerization, and histone methylation. Although it is necessary to elucidate the relationship of these functions with brain tumors and whether rare tumor mutations are enriched or tumor-unrelated mutations are selected in the PDX model, our study may call for the additional consideration of the model-specific genetic selection properties of PDX models.

## 2. Materials and Methods

### 2.1. Tumor Samples from Patients with Brain Tumors

**Figure 1:**
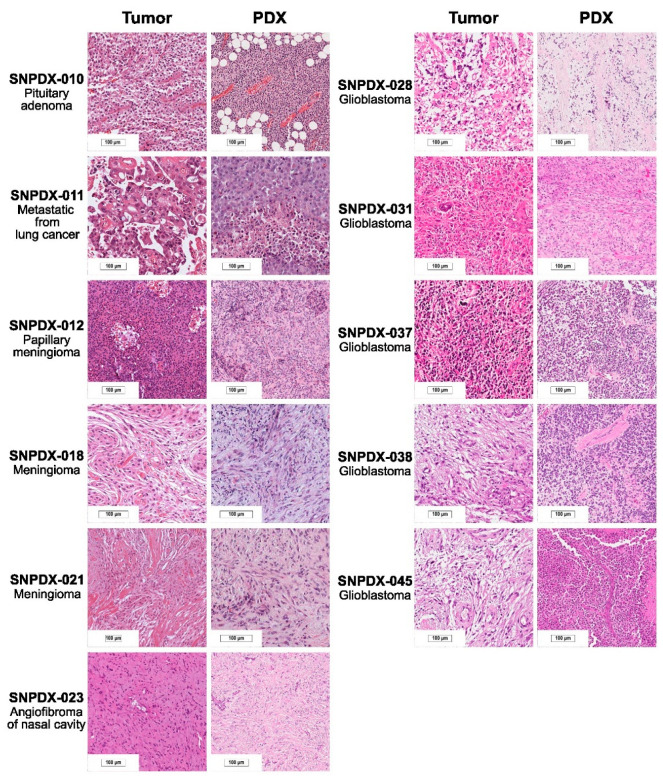
Histological examination of brain tumor–PDX tissues and their sources. We confirmed that the brain tumor type was maintained in the xenograft condition (Scale bar for 100 μm).

Tissue samples were obtained from ten patients with brain tumors between September 2015 and April 2017. This study was approved by the Institutional Review Board of Seoul National University Hospital (approval number H-1507-145-692), and all patients provided signed informed consent accordingly. The clinical diagnoses of the studied patients are provided in Figure 1 with representative histopathology images. In addition, clinical data such as age, gender, smoking status, stage, tumor size, preoperative chemotherapy, differentiation, vascular invasion, perineural invasion, lymphatic invasion, pleural invasion, recurrence, and survival were obtained from the patients’ medical records. Overall survival was defined as the time between histological diagnosis and death or the last follow-up. Relapse-free survival was defined as the time between histological diagnosis and the first progression or recurrence, death as a result of disease, or the last follow-up. Clinical information related to patients is provided in Supplementary Table S1.

### 2.2. Establishment of Brain Tumor–PDX Models

The tumor samples of patients were subcutaneously implanted into the flanks of NSG mice (Jackson Laboratory, Sacramento, CA, USA) to establish PDX models. The patient tumor tissue was obtained within two hours after surgical resection. Tumor tissue was implanted as soon as practical post-collection. The tumor was maintained at 4 °C in an appropriate storage medium (RPMI1 1640; Thermo Fisher Scientific, Waltham, MA, USA) until implantation. An anesthesia chamber was placed in the cabinet, and an oxygen/isoflurane mixture was prepared. Then we transferred fresh patient tissue to a culture dish and washed it out using serum-free RPMI 1640 media 1~2 times. The viable portion of the tumor was chosen to be diced into 1 mm^3^ pieces using a sharp blade. The surgical table and equipment were sterilized with 70% ethanol, and the surgical equipment (scissors, forceps, and cotton swabs) was autoclaved. Mice were put into the anesthesia chamber with an oxygen/isoflurane mixture. Minced tumor fragments were placed into a syringe with a trocar needle using small Iris forceps. The needle was inserted into the flank of a mouse using large straight forceps. The tumor fragments were engrafted using a plunger, and the needle was slowly removed while the engrafted tumor was held using forceps. The skin was disinfected with alcohol, and the mouse was transferred to a warm pad and observed until it awoke from anesthesia. Once the tumor reached 60 mm^3^ in volume, its size was measured using a caliper twice per week. The tumor volume was calculated as 0.5 × (length) × (width)^2^. When the tumor volume reached 600–800 mm^3^, the mice were euthanized, and the tumors were harvested for subsequent studies, such as successive passaging and next-generation sequencing analysis. The same harvested tissues were also used to prepare formalin-fixed paraffin-embedded blocks, and the remaining tissues were stored as snap-frozen tumor fragments. All animal care and experiments were performed under an animal protocol approved by the Biomedical Research Institute at Seoul National University Hospital (approval number: IACUC 14-0016-C0A0(1)).

### 2.3. Histopathological Analysis

Fresh patient and PDX tissue sections were cut at a thickness of 4 μm. Hematoxylin and eosin staining was performed using a Symphony system (Ventana Medical Systems, Inc., Basel, Switzerland) according to the manufacturer’s instructions. Images were analyzed with a ScanScope^®^ XT scanner (Aperio, Leica Biosystems, Newcastle, UK).

### 2.4. Mutational Analysis

The exon capture library of the samples was prepared using a TruSeq DNA Exome kit and sequenced with Illumina HiSeq 2000. Adapter sequences were trimmed using trimmomatic (version 0.39) [17] and mapped to the human–mouse combined reference genome (hg38 and mm10) using bwa (version 0.7.12) [18]. To remove reads from the mouse host in xenograft samples, the human (hg38) and mouse (mm10) genomes were compiled together and reads that preferentially mapped on mouse chromosomes were discarded. The same procedure was applied to human only tumor tissue and blood data for consistency. Somatic and germline variants were called using strelka2 (version 2.9.10) [19] with the ‘--exome’ option and considered passed variants for further analysis. The EnsEMBL Variant Effect Predictor (VEP) (version 101) [20] was used to predict the effect of somatic mutations, and genes with mutations assumed to have a high impact on the proteins were mainly analyzed. Cancer census genes and other genes associated with brain tumors were obtained from the COSMIC database (version 94) [21].

### 2.5. Mutational Signature Analysis

Somatic mutations in each sample were analyzed under single base substitution (SBS), double base substitution (DBS), and insertion-deletion (ID) signatures using the COSMIC MutSig reference database [22] and SigProfiler software (version 3.2—March 2021) [23].

### 2.6. Gene Ontology (GO) Term Enrichment Analysis

Genes with deleterious mutations were sorted based on the number of tumors or PDX samples in which the mutations were observed. GO term enrichment analysis was performed using PANTHER Gene List Analysis tools [24] with the PANTHER GO-slim biological process database. Enriched terms with an enrichment score greater than 3 and a false discovery rate less than 0.05 were selected.

## 3. Results

### 3.1. Deleterious Somatic Mutations Are Discordant between the Original Tumors and Xenograft Tissues

We established 13 PDX models using tumor tissues derived from ten brain cancer patients and confirmed the histopathological characteristics of the brain cancers and their derived xenograft tissues (Figure 1). Except for SNPDX-045 analyzed in the second passage, all PDX models were analyzed in vivo in the first passage, and SNPDX-010 and SNPDX-011 were additionally analyzed in the second passage. Then, we performed the whole-exome sequencing of these tissues and each patient’s blood to identify somatic mutations that accumulated in the brain tumors and to study their conservation in xenograft tissues. To focus on mutations that contribute to brain tumor development, we selected deleterious mutations with a high impact on protein functions, such as peptide truncation and loss of function induced by non-synonymous mutations, according to the prediction of the EnsEMBL VEP [20].

**Figure 2:**
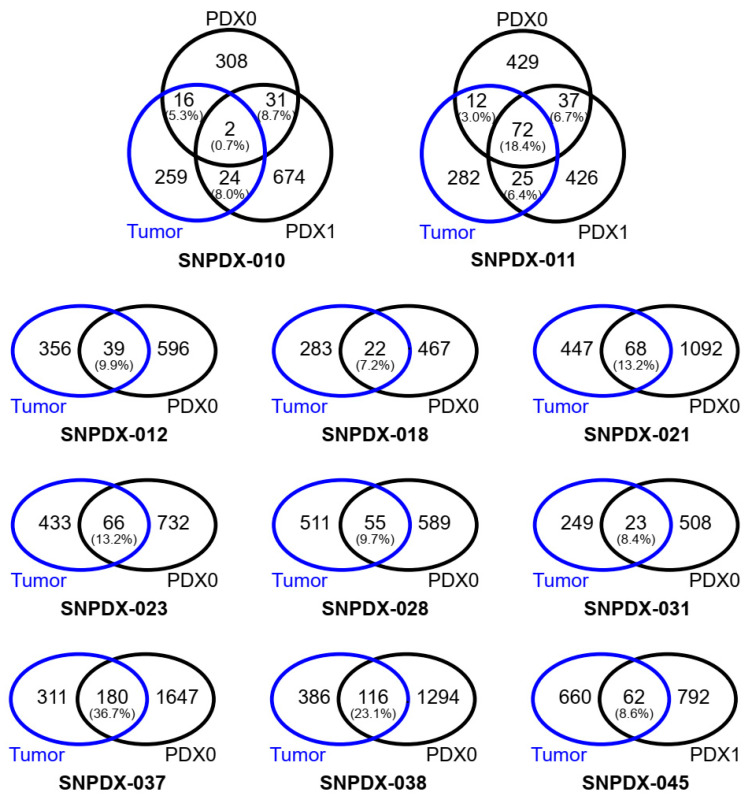
Overlap of deleteriously mutated genes between original tumors and PDX samples. The number of genes with deleterious somatic mutations was based on mutations with a high impact estimated by the EnsEMBL VEP. The proportion of overlapped genes between PDX samples and tumors was calculated as the number of overlapped genes divided by the number of genes in the original tumor. The proportion of overlapped genes between PDX0 and PDX1 (with additional passage) was calculated as the number of overlapped genes divided by the number of genes in the initial PDX sample (PDX0).

Surprisingly, the overlap of deleteriously mutated genes between tumors and derived xenograft tissues was much lower than expected (6.0–36.7%) because there were fewer deleteriously mutated genes in either sample (Figure 2). Moreover, even in the two cases whose xenograft tissues underwent a second passage (SNPDX-010 and SNPDX-011), the two xenograft tissues did not markedly share commonly mutated genes (SNPDX-011 had 18.4% of total mutated genes shared in all three samples at best). When we compared direct mutation sites rather than mutated genes, the overlap was even lower (Supplementary Figure S1) and was less than 3% in eight out of 11 cases. Even for samples with 21.2% overlap (SNPDX-037), overlap decreased to 5.2% if we considered the 2067 mutated genes observed in the xenograft tissue. Therefore, we concluded that the detailed somatic mutation profiles are unexpectedly not preserved between the original tumors and xenograft tissues.

### 3.2. Germline Variations Are Concordant between Original Tumors and Xenograft Tissues

Before evaluating the details of genetic selection in the host, we sought to confirm that global genetic features were shared among samples. If samples are collected from the same individual, germline variations should be highly overlapped. Therefore, we compared the germline variation of the PDX samples with that of each individual’s tumor and blood samples. In this analysis, we only considered homozygous germline variations to minimize the effect of the variant calling algorithm and sequencing errors.

**Figure 3:**
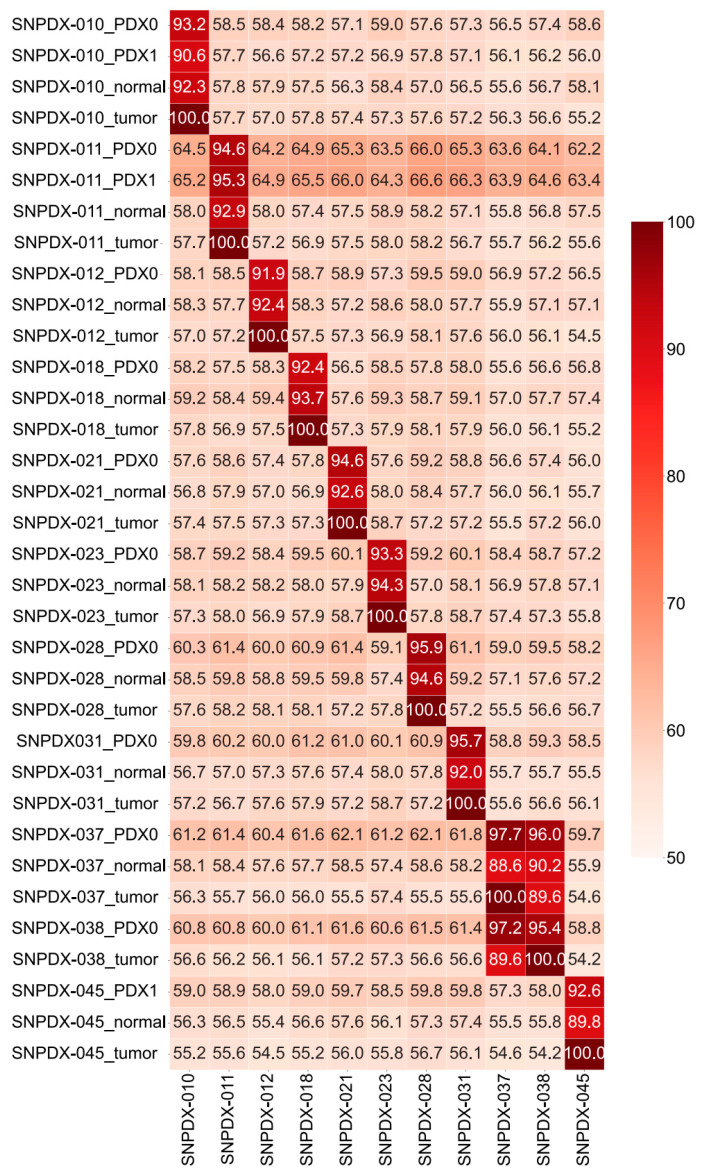
Heatmap of common homozygous germline variations. We only counted homozygous germline variants to compare the variations among samples. It is important to validate the concordance between tumors and xenograft samples; therefore, we used the number of germline mutations in a tumor sample, rather than a normal sample, as a base number for comparison. The overlapped percentages were calculated by dividing the number of germline mutations in xenograft samples by the number of germline mutations in tumor samples from each patient.

On average, 36,240 homozygous variants were identified in each sample, and more than 90% of variants overlapped among the blood, tumor, and PDX samples from the same individual (Figure 3). SNPDX-037 and SNPDX-038 were derived from two different tumor sites in the same patient and therefore their germline variations highly overlapped. By contrast, the overlapped proportion of homozygous variations among samples from different individuals did not exceed 60%, regardless of the sample type, except for the PDX samples derived from SNPDX-011, which had a 62–66% overlap. These results indicate that these PDX samples retained the genetic background of their matched tumor source and originated from the same tumor source.

### 3.3. Mutational Signatures Are Well Conserved between Tumors and Xenografts, and Provide Information about the Source of Mutations

The other global genetic features that were examined were mutational signatures, which can infer the source of somatic mutations in many different types of cancers [22]. We examined whether mutational alterations occurred during PDX establishment. Although the PDX models were well established from the same tumor source as described above, somatic mutational changes would be generated upon exposure to a novel mutagen during PDX establishment. Therefore, to analyze the mutational process in the PDX model and original tumor, we compared the mutational patterns between the PDX model and its matched original tumor using COSMIC mutational signature analysis.

**Figure 4:**
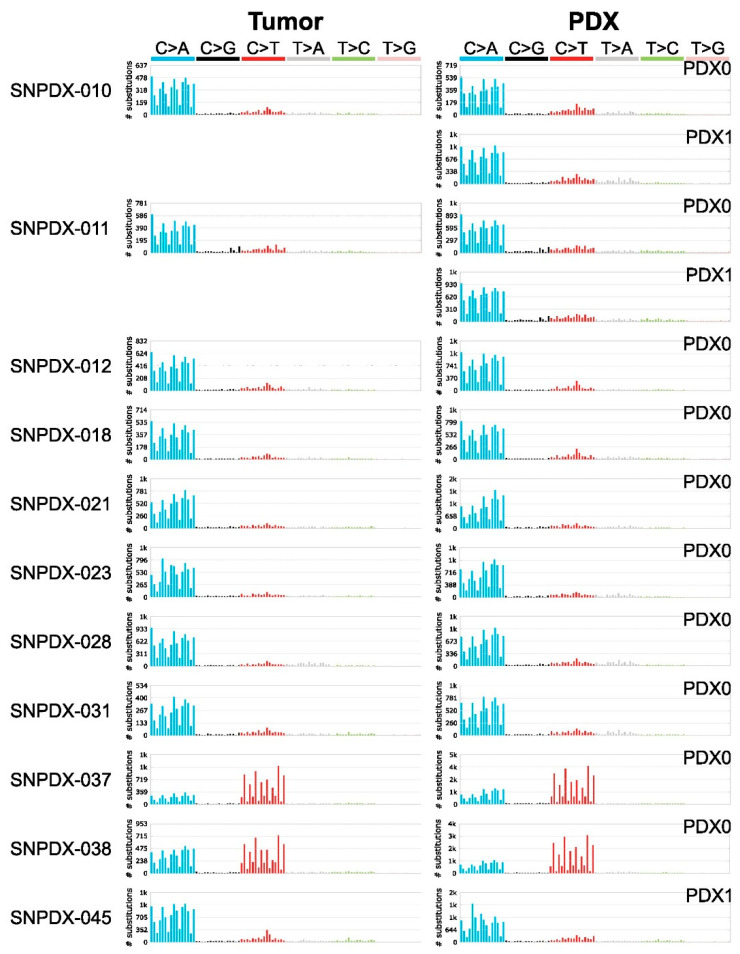
SBS mutational signature analysis. Mutational signatures were well conserved between tumor and xenograft samples. Mutational signatures derived from tumor and PDX samples were processed using COSMIC SBS signature analysis (see Methods for details). SBS mutational signatures of 96 subtypes from original tumors (**left**) and PDX samples (**right**) are represented. In addition, the mutational patterns of a xenograft that underwent a second passage (PDX1) in three patients (SNPDX-010, SNPDX-011, and SNPDX-045) are labeled in the plot.

All samples, except SNPDX-037 and SNPDX-038, had similar mutational patterns that were highly related to the SBS18 mutational signature previously introduced as possibly damaged by reactive oxygen species (Figure 4) [22,25,26]. Additionally, DBS and ID mutational signatures were conserved between the PDX samples and their matched tumors. Therefore, while somatic mutation profiles were highly discordant between the PDX samples and the original tumors, the PDX samples preserved the mutational signatures of their matched tumor samples. These results demonstrate that PDX models can retain not only the genetic background but also the in vivo mutational process of the original tumor.

As mentioned above, SNPDX-038 and SNPDX-037 were obtained from the same patient, and their mutational signatures were similar and were most like the SBS11 signature, which was reported to represent the temozolomide (TMZ)-related mutation signature (Figure 4) [22,27,28]. Indeed, the patient who provided SNPDX-037 and SNPDX-038 had undergone eight cycles of TMZ treatment for eight months before surgery. Taken together, these results demonstrate that PDX samples retain the mutational signatures of the original tumors, from which we can infer the cause of mutation, such as chemotherapy history.

### 3.4. Brain Tumor-Associated Mutations Are Enriched in PDX Samples

Although the PDX samples retained the mutational signatures and germline variations of the original tumors, their different somatic mutation profiles were puzzling. We speculated that this difference might be caused by a combination of the heterogeneity of tumors and their clonal evolution inside the host. Therefore, we examined the genes and their associated biological processes that were concordant and discordant in the tumors and the PDX samples.

**Figure 5:**
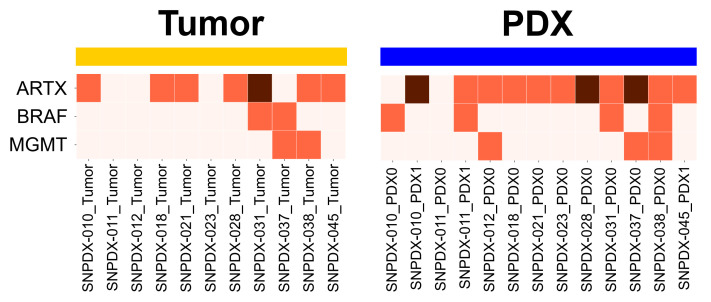
Mutation profile of previously identified genes related to brain tumor development. The list of genes was obtained from the COSMIC database, and the effect of deleterious somatic mutations was predicted as ‘MODERATE’ or ‘HIGH’ by the EnsEMBL VEP. Genes with HIGH-impact mutations are highlighted in a darker color, and MODERATE-impact ones in a lighter one.

We first examined the somatic mutation profiles of brain cancer-associated genes reported in the COSMIC database (Figure 5) [21]. Three genes (ARTX, BRAF, and MGMT) contained mutations with a moderate or high impact on protein functions in the original tumor or PDX sample. However, even in these brain tumor-associated oncogenes, similar to overall somatic mutations, we observed discordant mutation patterns between the tumors and PDX samples. Although the mutation profiles did not overlap, the PDX samples had more mutations in these genes than the tumors, indicating that brain tumor-associated mutations are enriched in PDX samples.

**Figure 6:**
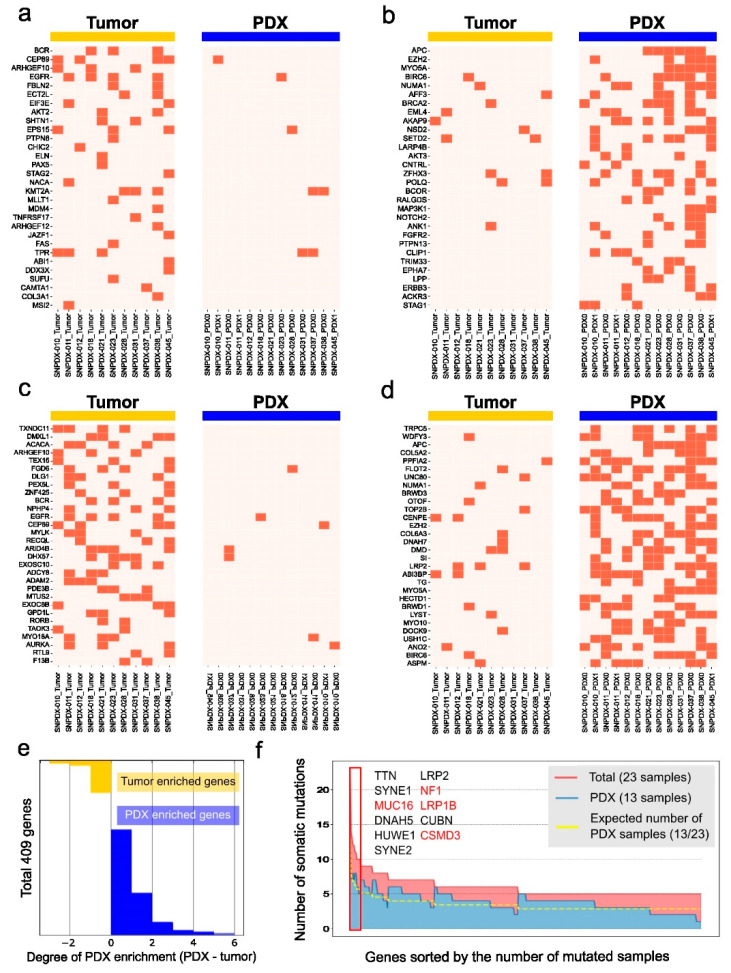
The top 30 ranked mutated genes enriched in tumors and PDX samples. We only considered deleterious mutations with a high impact on the alteration of protein function (presented with a orange color). (**a**) COSMIC census genes enriched in tumors. (**b**) COSMIC census genes enriched in PDX samples. (**c**) The top 30 ranked mutated genes out of 217 tumor-enriched mutated genes, (**d**) The top 30 ranked mutated genes out of 1610 PDX-enriched mutated genes. (**e**) We defined 409 genes with high-impact mutations enriched in PDX samples (blue) or original tumors (yellow) and presented the numbers of samples with these mutated genes in a bar graph. (**f**) Deleteriously mutated cancer genes were commonly observed in tumors and PDX samples. The yellow line is the expected number of PDX samples. This is lower than the actual number of PDX samples with mutated genes (blue area), indicating that these genes are enriched in PDX samples. Genes mutated in more than ten samples are listed (red box). Genes annotated as COSMIC census genes are marked in red.

We additionally investigated genes with somatic mutations enriched in the PDX samples or the original tumors. First, we examined 723 COSMIC cancer census genes commonly associated with various tumors (Figure 6a,b) and identified the top 30 genes enriched in either sample type. Surprisingly, compared with the mutated cancer census genes enriched in the tumors, many PDX-enriched mutated genes were shared in the PDX samples. We expanded this to all mutated genes and identified 217 tumor-enriched gene mutations and 1610 PDX-enriched mutations. Not only the number of genetic mutations but also the overlap among samples was higher in the PDX samples than in the tumors; therefore, we reasoned that tumor-associated mutations are enriched in PDX samples.

We defined 409 genes with high-impact mutations (such as protein truncation, frameshift mutation) and investigated whether these gene mutations were enriched in tumors or PDX samples by subtracting the number of tumor samples with the mutated genes from the number of PDX samples with the same mutated genes (Figure 6e). These gene mutations were enriched in the PDX samples compared with the tumors. We sorted these genes by the number of mutated samples and investigated whether the PDX samples had more cancer-associated mutations (Figure 6f). Based on the number of samples in each group, we expected 13 of the 23 samples with the given mutations to be PDX samples (yellow line in Figure 6f). The PDX samples had more mutations than the original tumors in most cases. When we focused on the 11 genes with high-impact somatic mutations in more than ten samples (red box in Figure 6f), the PDX samples had more mutations than the original tumors, and four of these genes (MUC16, NF1, LRP1B, and CSMD3) were annotated as COSMIC census genes. We concluded that cancer-associated somatic mutations are enriched in PDX samples, even though they are not detected in the original tumors.

### 3.5. Histone Methylation- and Cilium-Related Gene Mutations Are likely Enriched in PDX Samples

If brain tumor-specific mutations are enriched in PDX samples, we speculated that tumor-associated gene mutations that are not detected in the original tumor would be enriched in the PDX sample. To analyze enriched mutated genes in the tumor and PDX samples, we listed the individual enriched gene sets in each sample type. Genes with deleterious mutations were more diverse among the tumor samples, probably due to the heterogeneity of these samples.

**Figure 7:**
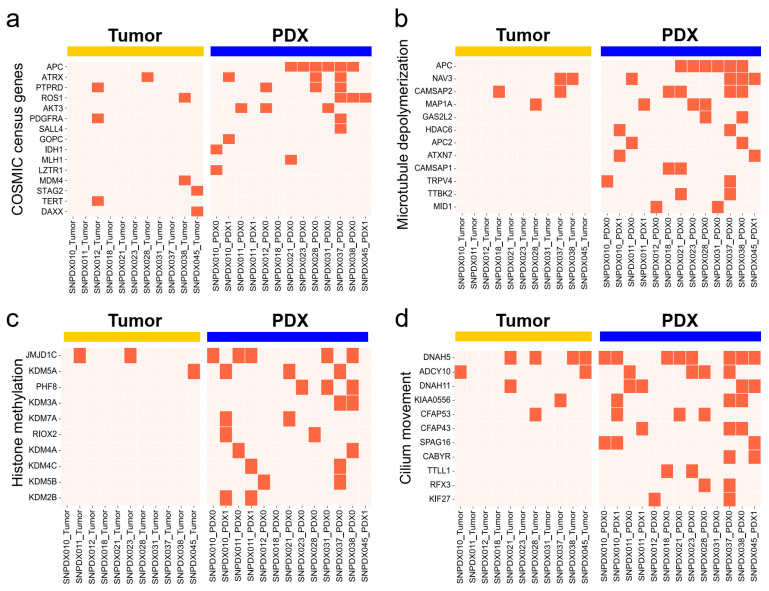
Histone methylation- and cilium-related genes are enriched in PDX samples. We only considered deleterious mutations with a high impact on the alteration of protein function (presented with a orange color). (**a**) Profile of deleteriously mutated genes among COSMIC cancer census genes. We only considered genes related to GBM and cancers in the CNS. (**b**–**d**) Genes associated with (**b**) histone methylation (GO:0016571), (**c**) microtubule depolymerization (GO:0007019), and (**d**) cilium movement (GO:0003341) were significantly enriched among PDX-specific enriched mutated genes.

While 761 genes were selected as tumor-enriched genes, GO term analysis of the enriched mutated gene set of original tumors found no significant terms, probably due to low sensitivity owing to tumor heterogeneity. By contrast, we identified 1610 genes enriched in the PDX samples, and GO terms related to histone methylation, cilium movement, and microtubule depolymerization were significantly overrepresented (Figure 7). These genes matched with those identified in a previous study, which reported genes involved in ciliary or flagellar motility [15]. These PDX-specific mutations associated with histone methylation, cilium movement, and microtubule depolymerization were not detected in the original tumors and might only be beneficial in the PDX environment. However, as described above, cancer-related genes had more mutations in the PDX samples than in the original tumors; therefore, it is also possible that rare tumor-related mutations are enriched in PDX samples. However, further research is required to investigate whether this is truly related to the pathology of brain tumors or preferential mutation in the PDX environment.

## 4. Discussion

PDX models have been widely used to investigate tumors because they can mimic the in vivo environment where tumors grow. The rapid proliferation of tumor cells can help enrich tumors in this environment, which can help identify tumor-related mutations and to study their characteristics. However, although key driver somatic mutations were claimed to be well conserved between the original tumors and the PDX samples, comprehensive somatic mutations across genomes were not thoroughly investigated.

Here, we analyzed the somatic mutation profiles of 13 PDX models from ten brain tumor patients and their original tumors. Even with this limited number of cases, the discordance of somatic mutations between the original tumors and the derived PDX samples showed that established PDX models may not recapitulate the genetic characteristics of the original tumors. Alternatively, insufficient genetic information might have been obtained owing to the high heterogeneity of brain tumors. Our sequencing depth was much lower (30×) than the recommended depth of whole-exome sequencing for the somatic mutation analysis of tumors (120×); therefore, it might be challenging to identify all somatic mutations in both sample types. A recent study suggests that patient-derived explants and glioma sphere lines derived from glioblastoma retain genetic similarity [29]. However, this method cannot guarantee that the enriched cell population represents the tumor in vivo because the selection condition used for in vitro culture can alter the heterogeneous tumor population.

The cohort in our study is composed of small numbers and diverse pathologies of various brain-associated tumors (five glioblastomas, two meningiomas, an angiofibroma of the nasal cavity, metastatic cancer, and a pituitary adenoma), which show different biological behavior, may be the limitation of this study. We tried to establish more samples for this study, but the success rate was only 22% (Supplementary Table S3). Although this number is relatively low compared to other types of cancer PDX models, another study with brain tumor PDXs also reported a similar graduation success rate, so that it might be an intrinsic issue of brain tumor samples. Also, because we used a heterotopic PDX model in the mouse flank rather than the orthotropic model in a similar anatomic region of the brain, our model may not be suitable for modeling the physiological characteristics of the original tumors. However, even with this limitation, we at least confirmed the pathological histology concordance (Figure 1) and the matched germline mutations (Figure 3) and somatic mutational signatures (Figure 4) between tumor and PDX tissues, so these resources would be informative to understand genetic alteration of the PDX model. Similar types of analyses could also be applicable to more orthotopic brain tumor PDX data when they become available in the future. It is also possible that de novo mutations are accumulated during PDX development, which is over nine weeks in most of our PDX models. However, further studies are required to understand this process, especially in the heterogeneous conditions of the xenograft host.

When we analyzed the somatic mutations of genes related to brain tumor development, such as ARTX, BRAF, and MGMT, we identified more mutations in the PDX samples than in the original tumors (Figure 5 and Figure 7a). Furthermore, when we analyzed the more frequently mutated genes in each group, we observed more consistent mutation profiles in the PDX samples than in the original tumors (Figure 6). We also compared the whole somatic mutations in each sample to the cancer-related mutations reported in the COSMIC database. We found that about 6% of the mutations overlapped with the known tumor mutations from each original tumor and PDX sample (Supplementary Table S2 and Figure S2). However, the central nervous system-related mutation is observed slightly more in the PDX model than in the original tumor, which might support our argument about the enrichment of tumor cells in the PDX model. Although we could not confirm it due to the limited sequencing depth and the small number of samples in this study, we speculated that the tumor cells were enriched in the PDX samples, which would explain why we identified more tumor-related somatic mutations in these samples than in the original tumors.

We further examined the somatic mutations of TP53 [30] and EGFR [31], two genes frequently mutated in glioma (Supplementary Table S2). TP53 is a well-known tumor suppressor that plays a central role in cellular stress signaling and is the most commonly found gene in human cancer [32]. Especially, the TP53 mutation is a hallmark of the early development of lower-grade astrocytic glioma [33]. Except for two samples in this study (SNPDX-018 and SNPDX-045), TP53 mutations were observed in most original tumors and the PDXs (Supplementary Table S2). Among them, the R273G mutation in SNPDX-011 and the R175H mutation in SNPDX-037/038 were observed in both the original tumor and the PDX. On the other hand, the mutation patterns of EGFR were quite diverse between the original tumor and the PDX (Supplementary Table S2). Only the P568L mutation found in 0.39% of all WHO grade III glioma patients and in 0.25% of all glioblastoma patients [34] was observed in both the original tumor and the PDX in SNPDX-031. Although tumor-related known mutations are enriched in the PDX, putative driver mutation patterns on TP53 and EGFR were not concordant between the original tumor and the PDX. However, it is unclear whether it is due to the limited detectability of this study for rare mutations by low sequencing depth or the clonal selection of these mutations in the heterotropic PDX model.

Previous studies showed the concordance of genome-wide functional features, such as copy number variations and gene expression patterns, between PDX samples and the original tumors. We also confirmed that the mutational signatures of each PDX sample were well matched with their original tumors. Additionally, two samples (SNPDX-037 and SNPDX-038) retained the mutational signatures caused by TMZ treatment. The patients who provided SNPDX-028 and SNPDX-045 had also been treated with TMZ, according to our records (Supplementary Table S1), but we did not observe the SBS11 signature in these samples. These patients had not received TMZ treatment within one year before biopsy; therefore, we speculated that the SBS11 signature associated with TMZ treatment was diminished. This suggests that our PDX models can be used to recapitulate the mutational process of the original tumors.

Gene mutations related to histone methylation and ciliary functions were enriched among the PDX samples. A recent study showed that glioma stem cells (GSCs) derived from a GBM patient exhibited the suppression of ciliogenesis, which led to self-renewal rather than differentiation [35]. When the cilia were restored, the GSCs started to differentiate and escaped from the self-renewal process. Therefore, the deleterious mutations related to ciliogenesis in our PDX samples may be advantageous for tumor cells to block their differentiation and promote their self-renewal for proliferation. Another study reported that glioblastoma-initiating cells (GICs) had stem cell-like chromatin features and exhibited the loss of H3K9me3 [36]. The elevation of the H3K9me3 level by histone demethylase knockdown induced the apoptosis of GICs, but not of differentiated cells. These results indicate that the deleterious mutations related to histone methylation in PDX samples may help to maintain GICs during PDX establishment.

Although our study demonstrated that the subpopulation of tumor cells might increase in our PDX models because of in vivo growth-promoting properties, further studies are required to investigate whether the mutations enriched in PDXs are important in the original brain tumors due to their associations with tumor-initiating cells or tumor stem cells. Alternatively, these mutations may increase cellular fitness in the xenograft environment, independently of their roles in tumor progression. Further analysis of more PDX models and paired primary tumor cell models, as well as single-cell level analysis, will help elucidate how to utilize and interpret the genetic features of PDX tumor models.

## Data Availability

The sequencing datasets generated during the current study are not publicly available due to patient privacy and legal issues.

## Supplementary Materials

The following supporting information can be downloaded at: https://www.mdpi.com/article/10.3390/biomedicines11112934/s1, Figure S1: Overlap of deleterious mutation sites between original tumors and PDX samples; Figure S2: Overlap of deleterious mutation sites between original tumors and PDX samples, identified in other CNS tumors; Table S1: Clinical information of the PDX donors; Table S2: Mutation profiles of TP53, EGFR between original tumors and PDX samples; Table S3: PDX graduation success rate in this study.
