# Supplementary figures and images for: Enrichment of Deleterious Mutated Genes Involved in Ciliary Function and Histone Modification in Brain Cancer Patient-Derived Xenograft Models
Source: Biomedicines. 2023 Oct 30;11(11):2934. doi: 10.3390/biomedicines11112934 (PMC10669283; doi:10.3390/biomedicines11112934)

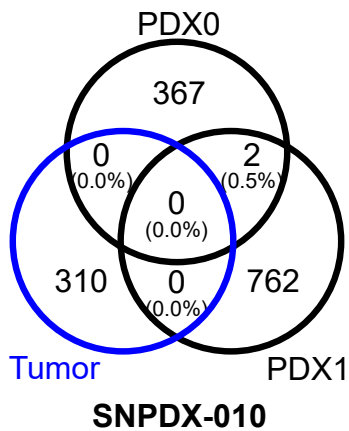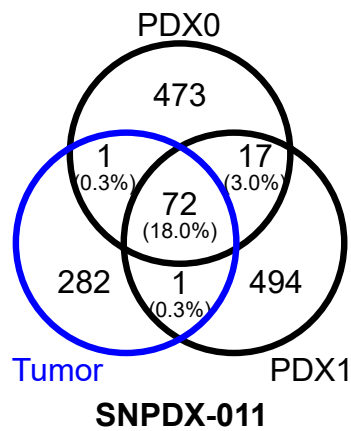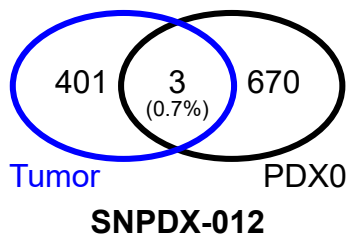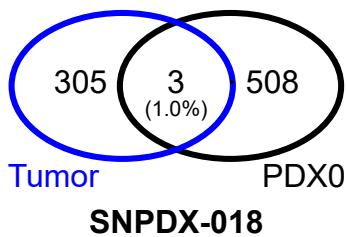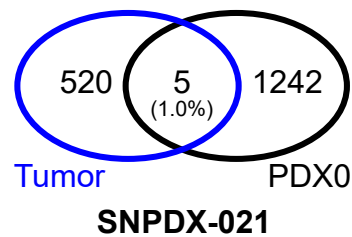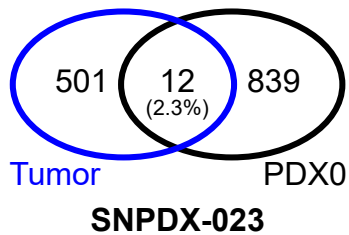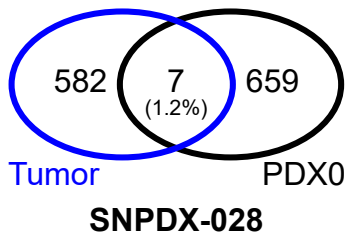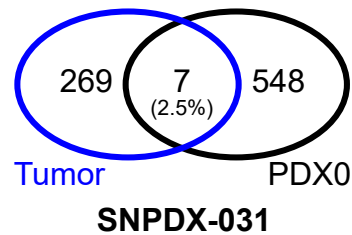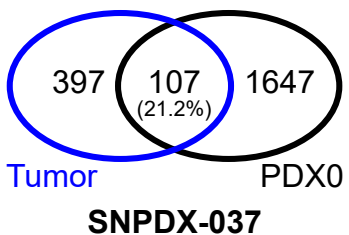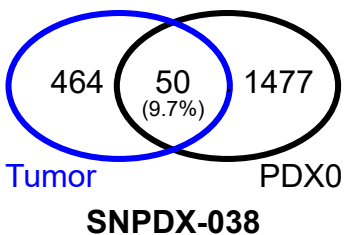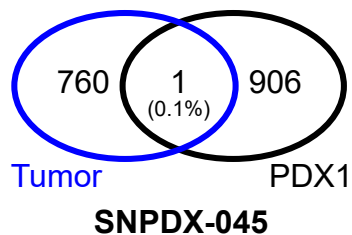

Supplement: Supplementary file 1 [file biomedicines-11-02934-s001.zip › biomedicines-2461238-supplementary/Supplementary.Final/SFig1.SomaticMutationVariation.v2.pdf]

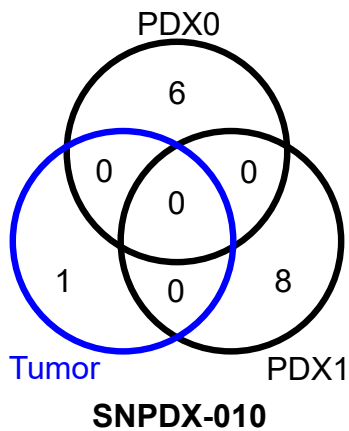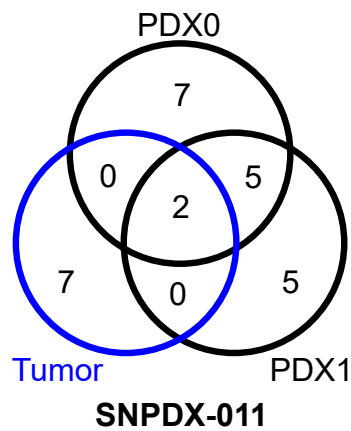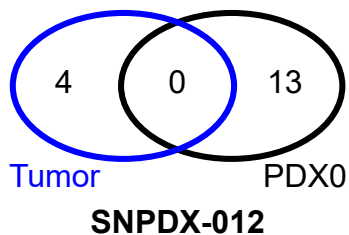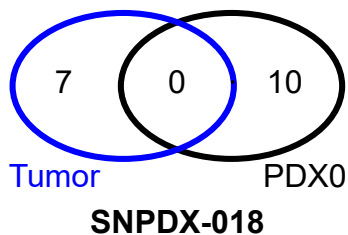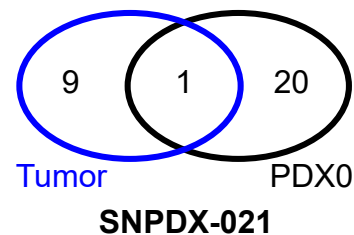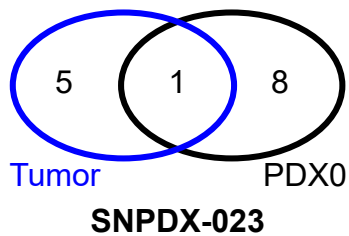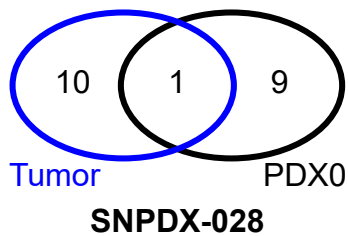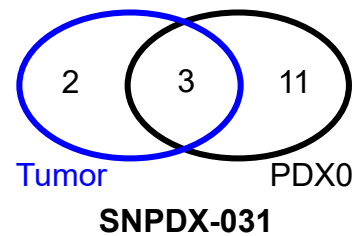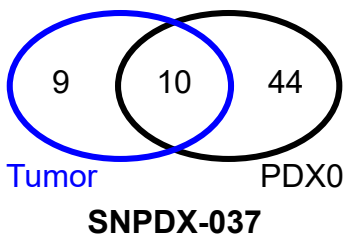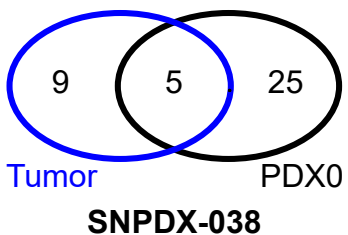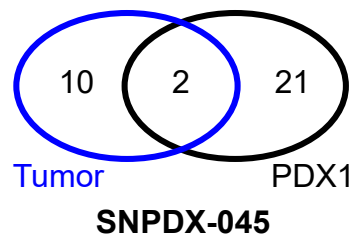

Supplement: Supplementary file 1 [file biomedicines-11-02934-s001.zip › biomedicines-2461238-supplementary/Supplementary.Final/SFig2.CNS_mutations.pdf]
